# Effects of Exogenous Yeast and Bacteria on the Microbial Population Dynamics and Outcomes of Olive Fermentations

**DOI:** 10.1128/mSphere.00315-16

**Published:** 2017-01-18

**Authors:** Jose Zaragoza, Zachary Bendiks, Charlotte Tyler, Mary E. Kable, Thomas R. Williams, Yelizaveta Luchkovska, Elaine Chow, Kyria Boundy-Mills, Maria L. Marco

**Affiliations:** Department of Food Science & Technology, University of California, Davis, California, USA; University of Minnesota

**Keywords:** fermentation, *Lactobacillus*, microbiota, resilient, *Saccharomyces*, spoilage, pectinases

## Abstract

In this study, we examined Sicilian-style green olive fermentations upon the addition of *Saccharomyces cerevisiae* UCDFST 09-448 and/or *Pichia kudriazevii* UCDFST09-427 or the lactic acid bacteria (LAB)* Lactobacillus plantarum* AJ11R and *Leuconostoc pseudomesenteroides* BGM3R. Olives containing *S. cerevisiae* UCDFST 09-448, a strain able to hydrolyze pectin, but not *P. kudriazevii* UCDFST 09-427, a nonpectinolytic strain, exhibited excessive tissue damage within 4 weeks. DNA sequencing of fungal internal transcribed spacer (ITS) regions and comparisons to a yeast-specific ITS sequence database remarkably showed that neither *S. cerevisiae* UCDFST 09-448 nor *P. kudriazevii* UCDFST 09-427 resulted in significant changes to yeast species diversity. Instead, *Candida boidinii* constituted the majority (>90%) of the total yeast present, independent of whether *S. cerevisiae* or *P. kudriazevii* was added. By comparison, *Lactobacillus* species were enriched in olives inoculated with potential starter LAB *L. plantarum* AJ11R and *L. pseudomesenteroides* BGM3R according to community 16S rRNA gene sequence analysis. The bacterial diversity of those olives was significantly reduced and resembled control fermentations incubated for a longer period of time. Importantly, microbial populations were highly dynamic at the strain level, as indicated by the large variations in AJ11R and BGM3R cell numbers over time and reductions in the numbers of yeast isolates expressing polygalacturonase activity. These findings show the distinct effects of exogenous spoilage and starter microbes on indigenous communities in plant-based food fermentations that result in very different impacts on product quality.

**IMPORTANCE** Food fermentations are subject to tremendous selective pressures resulting in the growth and persistence of a limited number of bacterial and fungal taxa. Although these foods are vulnerable to spoilage by unintended contamination of certain microorganisms, or alternatively, can be improved by the deliberate addition of starter culture microbes that accelerate or beneficially modify product outcomes, the impact of either of those microbial additions on community dynamics within the fermentations is not well understood at strain-specific or global scales. Herein, we show how exogenous spoilage yeast or starter lactic acid bacteria confer very different effects on microbial numbers and diversity in olive fermentations. Introduced microbes have long-lasting consequences and result in changes that are apparent even when levels of those inoculants and their major enzymatic activities decline. This work has direct implications for understanding bacterial and fungal invasions of microbial habitats resulting in pivotal changes to community structure and function.

## Abstract (precis)

Food fermentations are subject to tremendous selective pressures resulting in the growth and persistence of a limited number of bacterial and fungal taxa. Although these foods are vulnerable to spoilage by unintended contamination of certain microorganisms, or alternatively, can be improved by the deliberate addition of starter culture microbes that accelerate or beneficially modify product outcomes, the impact of either of those microbial additions on community dynamics within the fermentations is not well understood at strain-specific or global scales. Herein, we show how exogenous spoilage yeast or starter lactic acid bacteria confer very different effects on microbial numbers and diversity in olive fermentations. Introduced microbes have long-lasting consequences and result in changes that are apparent even when levels of those inoculants and their major enzymatic activities decline. This work has direct implications for understanding bacterial and fungal invasions of microbial habitats resulting in pivotal changes to community structure and function.

## INTRODUCTION

Fermented foods and beverages are the result of extensive microbial growth wherein microbial metabolism and biosynthesis pathways transform the starting food matrices (e.g., milk, fruits, and vegetables) to products that have distinct organoleptic properties. These fermentations are also dynamic ecosystems that provide the opportunity to investigate the ecological principles of invasion and succession (1). Modern industrial food fermentations frequently employ the use of starter cultures (e.g., certain breads, beer, cheeses, and yogurts) to initiate and control product quality (2). Traditional fermented foods generally rely on the microbial inoculants present on the raw materials or located on equipment and human handlers. However, for both approaches, spoilage defects can occur and lead to significant product and economic losses. A better understanding of the microbial community dynamics influenced by starter or spoilage microbe growth should result in informed approaches to manage fermentations for optimal sensory quality with minimal product defects.

Table olives are produced in quantities reaching approximately 2 million tons per year and are among the most important fermented fruits on the international food market (http://www.internationaloliveoil.org). Fermented table olives are both popular and nutritious and are commonly consumed in the Mediterranean diet (3). There are many different styles of fermented table olives because of the large number of tree varieties and processing methods available. The most common commercially produced fermented table olives processed in the United States are “natural green” and are referred to as “Sicilian-style.” These olives are harvested from Sevillano variety trees and then are submerged directly into acidified brine for 6 to 9 months. As for other olive fermentations, lactic acid bacteria (LAB) are essential for the transformation of the bitter and rigid olive fruits into palatable foods (4).

Although starter cultures are not yet used for the production of Sicilian-style olives in the United States, numerous studies have investigated strains of *Lactobacillus* and *Leuconostoc* as starter cultures for other olive fermentation methods (5–10). In those studies, strains of *Lactobacillus plantarum* and *Lactobacillus pentosus* were shown to be effective at increasing rates of brine acidification and improving the organoleptic qualities of the final product. However, examination of the effects of strain inoculants on the indigenous microbial community has been limited to either culture-dependent molecular methods (11–13) or global sequence-based measurements of the bacterial but not fungal taxa (7).

Similar to other fermented foods, fermented olives are also susceptible to defects and spoilage. Growth of contaminant bacteria such as *Bacillus* and *Clostridium* species can lead to gas pockets or the development of musty or putrid off-flavors (14). Spoilage can also be caused by molds and yeasts (14). Although yeasts are indigenous to many olive fermentations and contribute beneficially to texture and flavor development, these microorganisms are also associated with poor sensory qualities and damaged fruit (15). We recently found that pectinolytic yeasts indigenous to Sicilian-style fermentations are causative agents of extensive olive tissue damage and spoilage (4). Inoculation of* Saccharomyces cerevisiae* UCDFST 09-448, a strain with the capacity to degrade polygalacturonic acid, resulted in extensive structural losses to the olive mesocarp identical to those of spoiled olives produced at a commercial processor (4). By comparison, addition of *Pichia kudriazevii* UCDFST 09-427, a strain lacking polygalacturonase (PGA) activity, did not cause tissue damage.

A shared facet between starter and spoilage microbes is that they provide disproportionate contributions to the attributes of the final food product. These microbes are “invaders” of fermented food ecosystems. In this study, we investigated the effects of both types of organisms (starter and spoilage) on Sicilian-style fermented table olives. We measured the microbial community dynamics and textural/chemical properties of olives and brines that result from the addition of *S. cerevisiae* UCDFST 09-448, *P. kudriazevii* UCDFST 09-427, and starter cultures of *L. plantarum* and *Leuconostoc pseudomesenteroides*.

## RESULTS

### Changes to the acidity and texture of olives with LAB and yeast additions.

**FIG 1:**
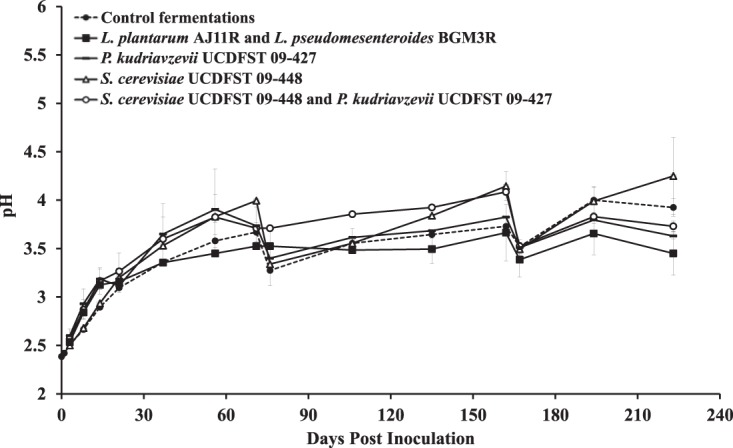
pHs of olive brines. The average ± standard deviation (SD) of two independent replicates at each time point is shown. Acetic acid (5.7 mM) was added to the brines of certain fermentations on day 76 (*S. cerevisiae* UCDFST 09-448, *P. kudriazevii* UCDFST 09-427, and the control fermentations) and day 167 (*S. cerevisiae* UCDFST 09-448, *P. kudriazevii* UCDFST 09-427, and those containing both *S. cerevisiae* UCDFST 09-448 and *P. kudriazevii* UCDFST 09-427).

The brine was at approximately pH 2.5 at the start of the study and increased over time (Fig. 1). Notably, the addition of the mother brine (pH 2.4) to all fermentations on day 38 did not alter the pH or titratable acidity. In order to maintain a pH conducive for fermentation, acetic acid was added to the brines with a pH above 4.0 at 76 and 167 days. The pH adjustment also changed the brine titratable acidity (see Fig. S1 in the supplemental material). Most of the fermentations requiring pH adjustment contained *S. cerevisiae* UCDFST 09-448 and/or *P. kudriavzevii* UCDFST 09-427. By comparison, LAB-inoculated fermentations tended to have the lowest pH over the course of the 225 days of study and were significantly more acidic than the controls from days 106 to 223 (*P* = 0.013 for the combined time point data, Mann-Whitney *U* test) (Fig. 1).

10.1128/mSphere.00315-16.1 FIG S1  Titratable acidity of olive brines. Titratable acidity was measured as % lactic acid (g/liter brine × 100%). Acetic acid (5.7 mM) was added to the brines of certain fermentations on day 76 (*S. cerevisiae* UCDFST 09-448, *P. kudriazevii* UCDFST 09-427, and the control fermentations) and day 167 (*S. cerevisiae* UCDFST 09-448, *P. kudriazevii* UCDFST 09-427, and those containing *S. cerevisiae* UCDFST 09-448 and *P. kudriazevii* UCDFST 09-427). The average ± SD from two independent replicates at each time point is shown. Download FIG S1, EPS file, 1.9 MB. Copyright © 2017 Zaragoza et al. 2017 Zaragoza et al. This content is distributed under the terms of the Creative Commons Attribution 4.0 International license.

The brine salinity decreased over time for all fermentations (see Fig. S2 in the supplemental material). NaCl was added periodically to maintain a salinity at or above 40 ppt and was increased to 60 ppt at the end of the study for consistency with commercial practices.

10.1128/mSphere.00315-16.2 FIG S2  Salinity of olive brines. At day 76, salinity was increased to approximately 45‰ in all treatments. Starting at day 173, the salinity was raised by 5‰ every 2 weeks until a final concentration of 60‰ was reached in all treatments. The average ± SD from two independent replicates at each time point is shown. Download FIG S2, PDF file, 0.1 MB. Copyright © 2017 Zaragoza et al. 2017 Zaragoza et al. This content is distributed under the terms of the Creative Commons Attribution 4.0 International license.

**FIG 2:**
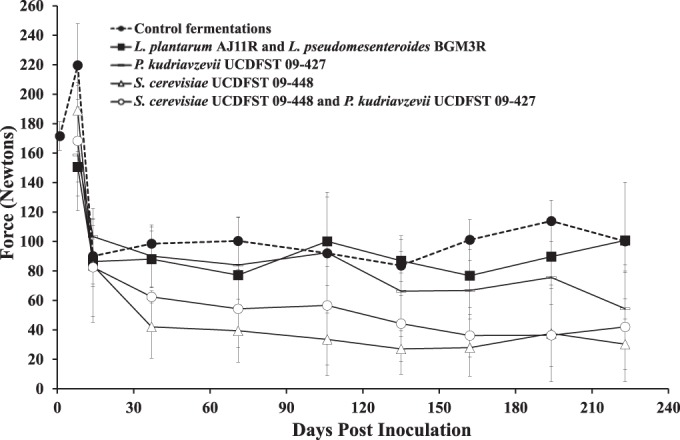
Texture analysis of olive firmness. The maximum force needed to displace olive tissue is plotted with respect to time after the initiation of the olive fermentations. Data points represent the average ± SD from 10 olives (5 from each of the replicate containers). Lower values indicate softer olives.

There were significant reductions in the firmness of all olives from an average of 17.9 N/g to 8.9 N/g between 8 and 14 days after submersion (*P* < 0.0001, Welch’s analysis of variance [ANOVA]) (Fig. 2). After 14 days, only the olives inoculated with *S. cerevisiae* UCDFST 09-448 continued to soften (Fig. 2) and therefore exhibited the spoilage defect observed previously (4). Those olives were approximately 2.4-fold softer than the controls, on average, at day 37 and remained significantly softer for the duration of the study (*P* < 0.0001, Kruskal-Wallis test). Although fermentations coinoculated with *P. kudriavzevii* UCDFST 09-427 and *S. cerevisiae* UCDFST 09-448 contained slightly firmer fruits compared to those inoculated with *S. cerevisiae* UCDFST 09-448 alone, this difference was not significant (*P* = 0.655, Mann-Whitney *U* test) (Fig. 2). In contrast, the firmness of olives treated as controls or inoculated with *L. plantarum* AJ11R and *L. pseudomesenteroides* BGM3R was similar to normal-quality commercially prepared olives.

### Growth of yeast in brines and olives.

**FIG 3:**
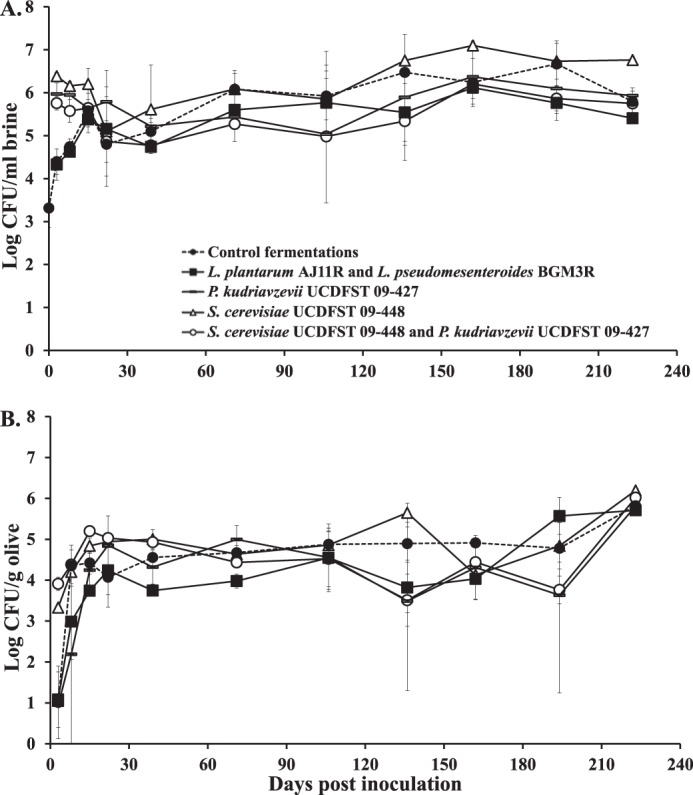
Yeast populations in pilot-scale Sicilian-style fermented olives and brines. Yeasts in brines (A) and associated with olives (B) were enumerated on RBCA culture medium throughout 7 months of fermentation. Each data point denotes the average ± SD (*n* = 2 for the brines and *n* = 6 olives [3 per replicate container]). The detection limits for yeast were 10^3^ CFU/ml in the brine and 10^2^ CFU/ml for the olives. The sampling dates were days 0 (after inoculation), 3, 8 to 9, 15, 22, 39, 71, 106, 136, 162, 194, and 223.

Brines inoculated with *S. cerevisiae* UCDFST 09-448 or *P. kudriavzevii* UCDFST 09-427 initially harbored approximately log 6.0 yeast CFU/ml (Fig. 3A). By comparison, the numbers of culturable yeast cells in control and LAB-inoculated containers at the start of the study were approximately 1,000-fold lower (Fig. 3A). Within 2 weeks, the levels of yeast in the brines were similar for all fermentations (Fig. 3A).

Olives harbored lower numbers of yeast than the brine (Fig. 3B). Olives from the controls and those inoculated with either *P. kudriavzevii* UCDFST 09-427 or LAB initially harbored less than 100 yeast CFU/g. Larger quantities of culturable yeast (between log 3 to log 4 yeast CFU/g) were recovered from the olives inoculated with* S. cerevisiae* UCDFST 09-448 and continued to increase for approximately the first 20 days (Fig. 3B). Subsequently, all fermentations tended to contain between log 4.0 and log 5.0 yeast CFU/g olive, and this number increased up to log 6.0 by day 225 (Fig. 3B).

### Proportions of yeast producing PGA in olive fermentations.

We measured for the presence of *S. cerevisiae* UCDFST 09-448 by screening individual yeast isolates from the olives and brine for their capacity to degrade polygalacturonic acid. Three days after the addition of yeast and LAB to the fermentations, between 80 and 100% of the colonies tested from containers inoculated with *S. cerevisiae* UCDFST 09-448 exhibited polygalacturonic acid (PGA) activity (Table 1). Sequence-based identification of those yeasts confirmed that the majority of the PGA-positive isolates were *S. cerevisiae* (data not shown). The levels of PGA-positive yeast in those fermentations remained high for at least 37 days. By comparison, much lower fractions (0 to 18%) of the yeast isolates from the other fermentations were PGA positive (Table 1). The highest numbers of yeasts with PGA activity in those fermentations were found 37 days into the study and encompassed 34% (48 PGA positive/143 total) and 36% (28 PGA positive/78 total) of the isolates from containers with either *P. kudriavzevii* UCDFST 09-427 or *L. plantarum* AJ11R and *L. pseudomesenteroides* BGM3R, respectively. Only 2 of the 209 yeast isolates tested from the control fermentations were able to degrade polygalacturonic acid. By 135 days, the number of yeast isolates with PGA activity was low in all fermentations (Table 1).

**TABLE 1:** Proportions of PGA-positive yeast

Treatment	Ratio (%) of PGA^+^ to total isolates at postinoculation day^a^:					
	3	8	14	21	37	135
Control fermentations	0/22	0/27	0/10	2/10 (20)	0/40	0/105
*L. plantarum* AJ11R and *L. pseudomesenteroides* BGM3R	2/25 (8)	3/19 (16)	5/14 (36)	7/38 (18)	28/78 (36)	3/39 (8)
*P. kudriavzevii* UCDFST 09-427	2/12 (17)	1/6 (17)	2/24 (8)	6/53 (11)	48/143 (34)	0/31
*S. cerevisiae* UCDFST 09-448	26/26 (100)	19/32 (59)	17/17 (100)	37/43 (86)	38/70 (54)	0/132
*S. cerevisiae* UCDFST 09-448 and *P. kudriavzevii* 09-427	27/32 (84)	17/24 (71)	33/38 (87)	32/40 (80)	50/85 (87)	0/22

a Shown is the ratio of PGA-positive isolates to total isolates recovered from both brine and olives.

### Bacterial cell numbers in brines and olives.

**FIG 4:**
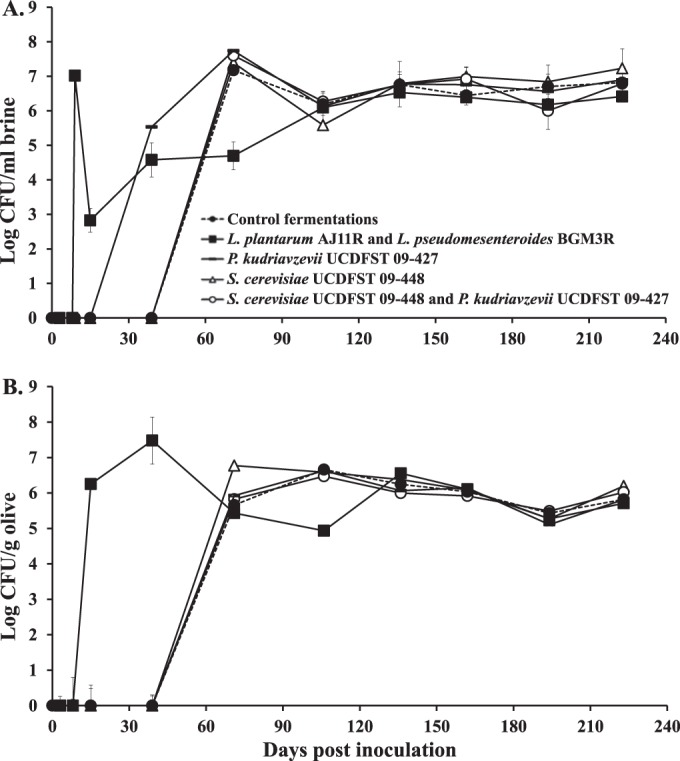
Bacterial populations in pilot-scale Sicilian-style fermented olives and brines. Bacteria in the (A) brines and (B) olives were enumerated on MRS. Each data point shows the average ± SD (*n* = 2 for the brines and *n* = 6 olives [3 per replicate container]). The detection limits for bacteria were 10^2^ CFU/ml in the brine and 10 CFU/ml for the olives. The sampling dates were days 0 (after inoculation to inoculation), 3, 8 to 9, 15, 22, 39, 71, 106, 136, 162, 194, and 223.

**FIG 5:**
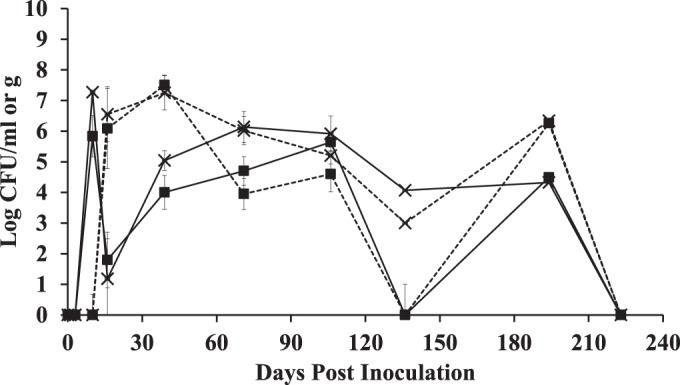
Numbers of *L. plantarum* AJ11 and *L. pseudomesenteroides* BGM3 cells in fermented olives and brines. Each point represents the total Rif^r^ bacterial numbers in brines (solid lines [log CFU per milliliter) and olives (dashed [log CFU per gram) from those fermentations inoculated with *S. cerevisiae* UCDFST 09-448 and *L. plantarum* AJ11 (×) and *L. pseudomesenteroides* BGM3 and *L. plantarum* AJ11 (■). Each data point is the average ± SD (*n* = 2 for the brines and *n* = 6 olives [3 per replicate container]). The detection limits were 10^2^ CFU/ml and 10 CFU/ml for the brines and olives, respectively. The sampling dates were days 0 (after inoculation to inoculation), 3, 9, 15, 22, 39, 71, 106, 136, 194, and 223.

For fermentations without added LAB, bacteria were first detected in the brine of the controls and containers inoculated with *P. kudriavzevii* UCDFST 09-427 at day 39 (Fig. 4A). Bacterial cell numbers were below the detection limit in fermentations containing *S. cerevisiae* UCDFST 09-448 (and no added LAB) until several weeks later. By comparison, the addition of *L. plantarum* AJ11R and *L. pseudomesenteroides* BGM3R resulted in the recovery of an average of log 7 CFU/ml brine immediately after inoculation on day 9 (Fig. 4A). Similar numbers were detected on MRS containing rifampin (MRS^Rif^), confirming the presence of the rifampin-resistant inoculants (Fig. 5). Six days later (day 15), the population sizes of bacteria in the brines of LAB-inoculated fermentations declined to log 3 CFU/ml on MRS (Fig. 4A) and log 1.5 CFU/ml on MRS^Rif^ (Fig. 5) and then increased again by the next time point (day 37).

Starting at day 106, all of the brines contained very similar bacterial population sizes, ranging from between log 6 to log 7 CFU/ml (Fig. 4A). Remarkably, after that time point, the numbers of the *L. pseudomesenteroides* BGM3R and/or *L. plantarum* AJ11R inoculants fluctuated dramatically according to viable cell counts on MRS^Rif^ and were below the detection limit by the end of the study (Fig. 5). These effects were not the result of the addition of the mother brine at 39 of the study, because no culturable bacteria on either MRS or tryptic soy agar medium were detected in the mother brine and yeast cells were detected at a low level (log 3.9 CFU/ml).

Bacterial population sizes associated with the olives followed similar patterns compared to those found in the brines (Fig. 4). By day 106, olives from all fermentations contained approximately log 6 bacterial CFU/g and remained at that level until the end of the study (Fig. 4B). Rif-resistant *L. plantarum* AJ11R and *L. pseudomesenteroides* BGM3R were associated with the olives on day 16, the same time point when the brines contained the lowest bacterial numbers (Fig. 5). The levels of culturable BGM3R and AJ11R associated with the olives then fluctuated after the total LAB quantities stabilized after day 106 (Fig. 4B; Fig. 5).

### Yeast and bacterial diversity in fermenting olives.

Microbial diversity in the olive fermentations was examined by high-throughput DNA sequencing of the fungal internal transcribed spacer (ITS) region 1 and V4 region of bacterial 16S rRNA genes. Because the majority of observed changes in microbial numbers occurred within 90 days after the olives were submerged (Fig. 3; Fig. 4), we focused on the olive-associated microbiota prior to that point.

**FIG 6:**
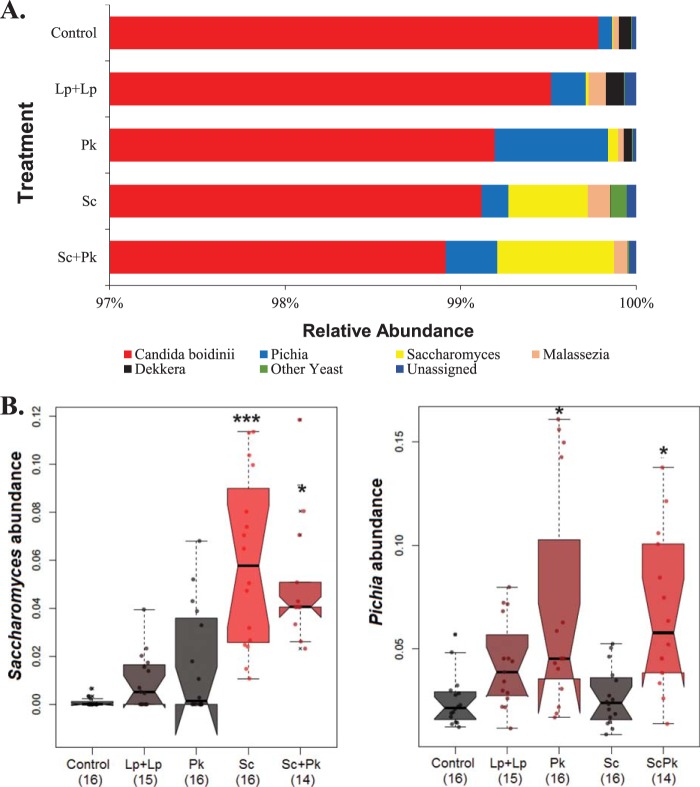
Proportions of *Saccharomyces* and *Pichia* in olive fermentations. (A) Relative abundance of all yeasts averaged across all time points for each fermentation. Displayed on the *y* axis are the different inoculants into the olive fermentations as follows: control, *L. plantarum* AJ11R and *L. pseudomesenteroides* BGM3R (Lp+Lp), *P. kudriavzevii* UCDFST 09-427 (Pk), 10^7^ CFU/ml *S. cerevisiae* UCDFST 09-448 (Sc), and *S. cerevisiae* UCDFST 09-448 and *P. kudriavzevii* UCDFST 09-427 (ScPk). (B) Multivariate analysis was performed using MaAsLin. The *y* axis displays the abundance of *Saccharomyces* (left) and *Pichia* (right) for fermentations described in panel A. ***, *P* < 10^−9^; *, *P* < 10^−4^.

Overall, the fungal populations associated with the olives were highly homogeneous and consisted of only a few yeast species. For the 6,167,514 ITS sequences retained after quality filtering, there were no significant differences between the fungal communities at different sampling times according to alpha and beta diversity metrics (observed operational taxonomic units [OTUs] and Bray-Curtis dissimilarity [data not shown]). In total, 27 OTUs were identified and distributed among eight different taxa. The most abundant taxon was *Candida boidinii* from the *Ogataea* clade, which comprised 99.4% of all ITS sequences (Fig. 6A; see Fig. S3 in the supplemental material). *Pichia* and *Saccharomyces* were also detected, and while recovered in low proportions from all olives, they were significantly increased in fermentations inoculated with *S. cerevisiae* UCDFST 09-448 and/or *P. kudriavzevii* UCDFST 09-427 (Fig. 6B). In fermentations inoculated with *S. cerevisiae* UCDFST 09-448, sequences classified as *Saccharomyces* reached a maximum abundance of 1.3% by day 37 (Fig. S3). For olives exposed to *P. kudriavzevii* UCDFST 09-427, *Pichia* sequences reached at an average abundance of 21.7% on day 8, and those percentages dropped to an average of 1.3% by day 21 (Fig. S3). When both *S. cerevisiae* UCDFST 09-448 and *P. kudriavzevii* UCDFST 09-427 were added, there was a higher relative abundance of *Pichia* on day 8 and there were higher proportions of *Saccharomyces* on day 21. Members of both genera fell to below 1% abundance in all fermentations by day 71 (Fig. S3).

10.1128/mSphere.00315-16.3 FIG S3  Proportions of yeast taxa in olive fermentations. Displayed is the relative abundance of yeast taxa associated with the olives. The *x* axis shows the treatment group and the specific day that the sample was taken after the start of the study. Download FIG S3, PDF file, 0.1 MB. Copyright © 2017 Zaragoza et al. 2017 Zaragoza et al. This content is distributed under the terms of the Creative Commons Attribution 4.0 International license.

**FIG 7:**
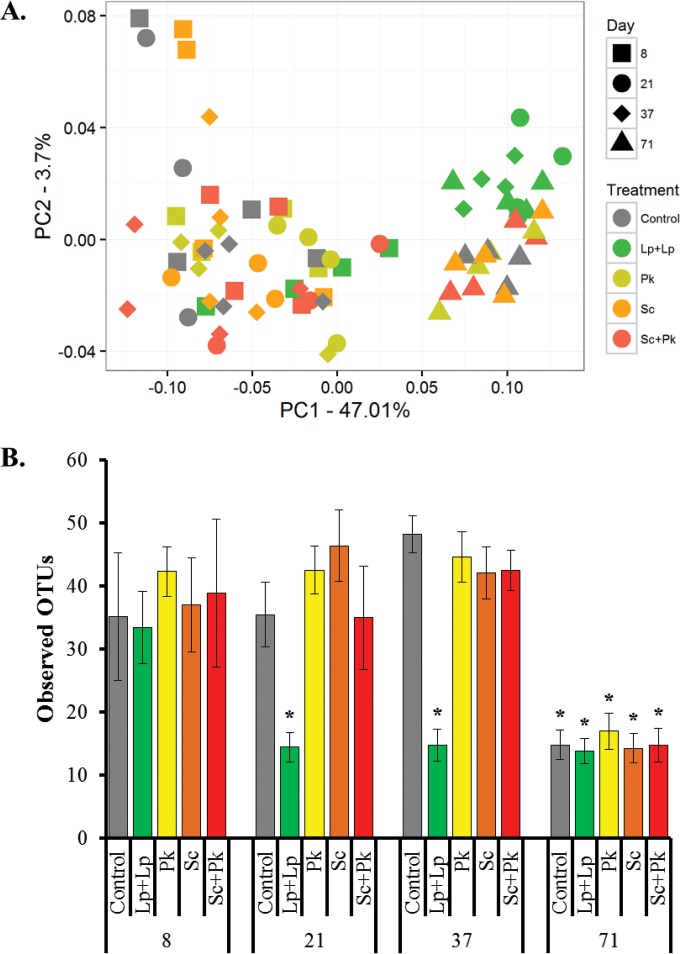
The effect of time and microbial inoculation on bacterial diversity. (A) Principal coordinate analysis (PCoA) of the weighted UniFrac (beta-diversity) distance metric. (B) Observed OTUs by day at a depth of 504 DNA sequences per sample. The average ± SD of the number of OTUs per time point per fermentation type is shown. Pairwise comparisons of observed OTUs between and within the treatments at different days were calculated using the Students *t* distribution test with FDR correction. Asterisks indicate conditions where the observed OTUs were significantly reduced compared to the control samples collected on day 8.

In contrast to fungi, bacterial populations underwent substantial alterations during the early phases of the olive fermentations. Principal coordinate analysis (PCoA) of the weighted UniFrac distance between microbial communities showed that olive-associated bacterial populations changed compositionally over time (Fig. 7A). For all of the fermentations, bacteria associated with olives at day 71 differed from those at earlier time points. Contributing to these differences were significant reductions in the richness of bacterial taxa present on the olives (*P* < 0.005, *t* test with false discovery rate [FDR] correction) (Fig. 7B).

**FIG 8:**
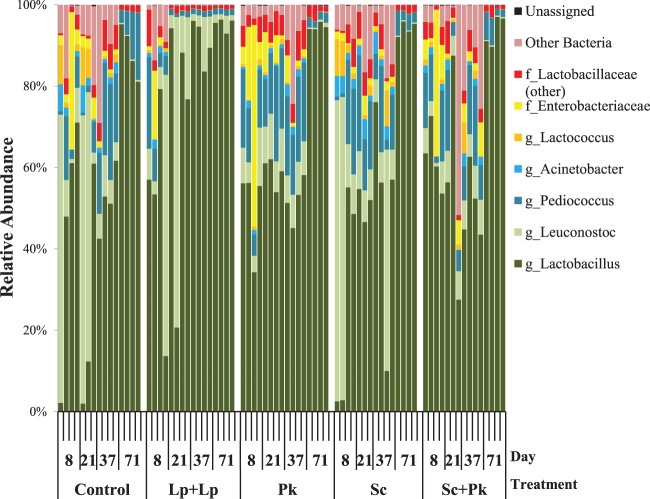
Bacterial composition of olive fermentations is affected by microbial inoculation. The relative abundance of 16S rRNA gene sequences classified at different taxonomic levels is shown for 3 to 4 olive samples for each time point and fermentation type. Olive OTUs with a relative abundance below 0.5% across the data set were grouped into the category “Other Bacteria.”

A total of 96 OTUs and 36 distinct bacterial taxa were found among the 1,424,999 DNA sequences examined. The majority of these sequences were assigned to LAB genera, including *Lactobacillus*, *Leuconostoc*, *Pediococcus*, and *Lactococcus* (Fig. 8). Other bacterial taxa, including members of the *Proteobacteria*, were also detected, but their proportions declined to background levels before day 71 (Fig. 8). Members of the *Lactobacillus* genus were most abundant, and among the 18 OTUs classified to this genus, 14 significantly increased in relative abundance from days 37 to 71 (FDR-corrected fitZIG *P* values of <0.0002). The *Lactobacillaceae* family, and particularly *Lactobacillus* and *Pediococcus*, comprised over 99% of all bacteria in the fermentations by day 71 (Fig. 8).

Bacterial populations on olives inoculated with *L. plantarum* AJ11R and *L. pseudomesenteroides* BGM3R were different from those in the control and yeast-inoculated fermentations. Before the addition of *L. plantarum* AJ11R and *L. pseudomesenteroides* BGM3R on day 9, species richness associated with those olives was comparable to those of the other fermentations. By day 21, significantly fewer OTUs were detected in association with the LAB-inoculated olives compared to those with *S. cerevisiae* UCDFST 09-448 and/or *P. kudriavzevii* UCDFST 09-427 (*P* < 0.005, *t* test with FDR correction) (Fig. 7B). At day 37, olives to which *L. plantarum* AJ11R and *L. pseudomesenteroides* BGM3R were added contained significantly fewer OTUs than all other fermentations tested (*P* < 0.005, *t* test with FDR correction) (Fig. 7B). The bacterial communities within these containers were dominated by *Lactobacillus* (Fig. 8). Notably, the bacterial diversity associated with olives from the *L. plantarum* AJ11R and *L. pseudomesenteroides* BGM3R fermentations at days 21 and 37 clustered with the other fermentations at day 71 (Fig. 7A).

## DISCUSSION

The introduction of exogenous spoilage yeast and starter LAB into Sicilian-style olive fermentations caused significant but distinct alterations to the numbers and diversity of microbes associated with the olives and brines. Yeast populations were only modestly and transiently affected when olives were inoculated with pectinolytic *S. cerevisiae* and/or nonpectinolytic *P. kudriavzevii*. However, this exposure was still sufficient for extensive softening and spoilage of olives containing *S. cerevisiae* UCDFST 09-448. Conversely, fermentations containing added *L. plantarum* and *L. pseudomesenteroides* strains exhibited significant increases in LAB and global changes in bacterial community composition that corresponded with increased brine acidity.

Similar to other studies on natural green olives (16), yeast numbers increased rapidly and were sustained in the control fermentations. Yeast cells were approximately 10-fold more abundant in the brines as opposed to the olives, as we found previously (4). Those olive yeast communities were dominated nearly exclusively by *C. boidinii*. *Candida*, and specifically *C. boidinii*, is a common inhabitant of olive fermentations, including those that are “natural green” (4, 17–19). Although *C. boidinii* was not as prominent in those fermentations as was found here, this distinction might be the result of our more thorough, culture-independent analysis of the yeast populations. Alternatively, because we only measured the microbiota for the first 71 days after olive submersion, it is possible that the dominant yeast species could have changed over time and subsequently during product storage (20).

Introduction of *S. cerevisiae* UCDFST 09-448 and *P. kudriavzevii* UCDFST 09-427 resulted in elevated numbers of yeast cells in the brine, strongly indicating that both strains survived, at least initially, in the fermentations. Olives inoculated with those strains also harbored significantly higher proportions of *Saccharomyces* and *Pichia* compared to the other fermentations when measured at days 21 and 37 according to community-level DNA sequence analysis. Notably, however, these genera constituted only between <1% and 42% maximal abundance in any of the olives tested at those time points. The proportions of these two genera then diminished to background levels within the first 70 days of the experiment. Thus far, few published reports have investigated the effects of exogenous yeast cultures on the population microbial dynamics and diversity in olive fermentations (5, 7, 16). According to culture-dependent methods applied in those reports, levels of the inoculated yeast cells were either maintained (5) or significantly reduced (16) as the fermentations progressed. Such variation might be the result of the processing method applied or yeast strain- or species-dependent differences in the capacity to successfully invade and colonize olive drupes in the presence of the indigenous fermentation-associated microbiota.

Even though fermentations exposed to *S. cereviseae* UCDFST 09-448 contained low proportions of this species, the mesocarp tissue integrity of those olives was significantly compromised. The observed losses in tissue firmness were also in agreement with the initially high proportions of PGA-positive yeasts in fermentations containing *S. cereviseae* UCDFST 09-448. Detrimental reductions in tissue firmness occurred approximately 30 days into the study. Even though softening was delayed for olives coinoculated with nonpectinolytic *P. kudriavzevii* UCDFST 09-427, this difference was not sufficient to prevent *S. cerevisiae* UCDFST 09-448-induced spoilage. Importantly, for all fermentations containing *S. cerevisiae* UCDFST 09-448, PGA-positive yeast cells were below the limit of detection by the end of the study. These results indicate that either olives are most susceptible to contaminant spoilage yeast during the early stages of fermentation prior to the development of stable yeast communities, or alternatively, that there is a finite time frame in which *S. cerevisiae* UCDFST 09-448 is able to successfully compete with the indigenous yeast microbiota.

Notably, the proportions of yeast species according to ITS sequencing differed compared to the high numbers of PGA-positive yeast cells detected in the *S. cerevisiae* UCDFST 09-448 fermentations. Although the significance of this distinction is not yet known, the disparity could be due to the presence of dead but intact *C. boidinii* in the fermentations or the result of viable but nonculturable cells that were unable to grow on the laboratory culture medium used here. For the latter, other yeast species were also found to enter into nonculturable states (21, 22). Ultimately, refinements to yeast-specific DNA sequencing methods combined with other techniques such as fluorescent *in situ* hybridization (FISH) will help to resolve this discrepancy and can be used for more thorough measurements of yeast communities than culture-dependent molecular typing methods which are of limited throughput and both costly and time-intensive. The value of culture-independent DNA sequencing has already been shown in measurement of bacterial communities associated with olive fermentations (7, 23), and therefore the yeast-specific ITS database developed here provides necessary and complementary information.

The populations initially associated with the olives were diverse, and up to 30% of the bacteria detected were non-LAB species. Growth of bacteria in the fermentations took between 3 and 5 weeks longer than found previously (4), possibly because of the lower starting pH (pH 2.5 as opposed to pH 3.0) of the brines used in the present study. The initial recalcitrance of the fermentations to LAB growth was also evident by our inability to recover culturable *L. plantarum* AJ11R and *L. pseudomesenteroides* BGM3R inoculants after their first addition to the brine. By day 9, the pH of all fermentations increased to 3.1, and our attempt at inoculating the LAB strains on that day was successful. Overall, the exogenous LAB resulted in significant modifications to the fermentations, including initial increases in total bacterial cell numbers and a more rapid progression toward LAB-dominated bacterial communities. To this point, the bacterial composition of olives exposed to *L. plantarum* AJ11R and *L. pseudomesenteroides* BGM3R for 3 weeks resembled the low-diversity, *Lactobacillus-*dominated microbiotas of olives that had fermented for over 2 months. This finding agrees with previous studies showing that LAB starter cultures can reach high numbers following inoculation into olives (11, 12) and inhibit competing microbes (24). Moreover, these additions changed the environment of the fermentations and, likely as a result of their production of organic acids, conferred consistently lower brine pH.

Remarkably, even with the lower bacterial diversity and favorable increase in the fermentation environment in olives containing *L. plantarum* AJ11R and *L. pseudomesenteroides* BGM3R, those fermentations were not static. The numbers of rifampin-resistant bacteria fluctuated significantly when measured after day 106 and were sufficiently low that they were below the limit of detection in the brines and olives at the end of the study. The lack of stability for individual strains within the fermentations was also noticeable among the yeasts, as indicated by the reductions in PGA-positive yeast over time, even in containers with *S. cerevisiae* UCDFST 09-448. To fully elucidate microbial community dynamics in food fermentations, it is therefore important to monitor the numbers of starter culture strains rather than rely on total (culturable) cell quantities. This approach should ultimately improve our understanding of the microbial succession and equilibrium dynamics that occur, even in relatively simple food fermentations.

Recent studies have provided evidence of interdomain interactions between yeast and LAB cocultures (25–27). Although we found no indication that the addition of spoilage yeast affected LAB species diversity (and vice versa), LAB were detected earlier in olives containing the added *P. kudriavzevii* UCDFST 09-427. This finding supports other studies on olives showing that at least certain yeast strains can have direct quantitative effects on LAB, potentially by promoting the release of nutritive compounds (15). Conversely, the addition of LAB did not have an apparent effect on total yeast numbers or diversity.

In conclusion, this study shows the significant but remarkably different effects that exogenous spoilage yeast and starter culture LAB have on the microbial community dynamics and outcomes of fermented foods. These strain additives, although potentially minor constituents of the food microbiota, can still have significant effects on related and unrelated species, including those in different domains of life (fungi and bacteria). This awareness adds to our understanding of the effects of microbial strain invasions on community stability in food ecosystems. The findings can be applied to improve selection methods for starter cultures and the design of microbial-based strategies that prevent defects by spoilage yeast.

## MATERIALS AND METHODS

### Microorganisms and culture preparation.

*S. cerevisiae* UCDFST 09-448, *P. kudriavzevii* UCDFST 09-427, *L. plantarum* AJ11, and *L. pseudomesenteroides* BGM3 were initially isolated from commercial Sicilian-style olive fermentations in California. The yeast strains are maintained in the Phaff Yeast Culture Collection (UCDFST; http://phaffcollection.ucdavis.edu/). Both *S. cerevisiae* UCDFST 09-448 and *P. kudriavzevii* UCDFST 09-427 were described previously (4). *L. plantarum* AJ11 and *L. pseudomesenteroides* BGM3 were selected because of their elevated acid, salt, and heat tolerance levels compared to other olive-associated, LAB isolates (data not shown). To enable the detection of *L. plantarum* AJ11 and *L. pseudomesenteroides* BGM3 in olive fermentations, spontaneous rifampin (Rif)-resistant mutants of these strains were selected after growth in de Man-Rogosa-Sharpe broth (BD, Franklin Lakes, NJ) containing 50 µg/ml Rif (MRS^Rif^) (Thermo Fisher Scientific, Waltham, MA). The rifampin variants were renamed BGM3R and AJ11R.

For inoculation into pilot fermentations, *S. cerevisiae* UCDFST 09-448 and *P. kudriavzevii* UCDFST 09-427 were grown in yeast mold (YM) broth (BD, Franklin Lakes, NJ) at 30°C with aeration for 48 h. *L. plantarum* AJ11R and *L. pseudomesenteroides* BGM3R were grown in MRS at 30°C without aeration for 24 h. The yeast and LAB cells were then collected by centrifugation at 15,300 × *g* for 10 min at 4°C and washed twice in saline (0.85% NaCl). Yeast numbers were adjusted to 10^8^ cells/ml and LAB to 10^9^ cells/ml with a hemocytometer (Bright Line, NY, USA) prior to inoculation into the olive brines.

### Olive fermentations.

Freshly harvested Sevillano olives were provided by a commercial California olive processor in October 2012 and submerged in 1-gal (3.8-liter) plastic containers containing brine (pH 2.5) with 5% NaCl and 0.3% lactic acid consistent with California industry practice. The olives lacked visible defects and did not show mesocarp damage. The containers were equipped with meshed plastic discs to ensure olive submersion. Lids contained pin-sized holes to permit ventilation. Within 24 h after olive submersion, the brine was inoculated to reach an estimated 10^7^ cells/ml of *S. cerevisiae* UCDFST 09-448, *P. kudriavzevii* UCDFST 09-427, *S. cerevisiae* UCDFST 09-448, and *P. kudriavzevii* UCDFST 09-427 together or *L. plantarum* AJ11 and *L. pseudomesenteroides* BGM3. To enable comparisons among the fermentations, control containers were inoculated with a similar volume of sterile physiological saline (95 ml). Because viable rifampin-resistant bacteria were not detected 24 h after inoculation according to growth on MRS^Rif^, these strains were added again on day 9 of the study. On day 38, commercial “mother brine” from 2011 was added to a final concentration of 0.2%. All of the fermentations were performed in duplicate and incubated in a climate-controlled room at 21 to 23°C. Salinity, pH, and titratable acidity were measured periodically as previously described (4). Salinity and acidity were periodically increased in the fermentations using NaCl and 5.7 M acetic acid, respectively, as recommended by the commercial processor.

### Texture analysis.

Olives were weighed and then measured for firmness with a TA-XT2 texture analyzer equipped with Texture Expert Exceed software version 2.64 and a TA30 compression probe (Stable Micro Systems, Godalming, United Kingdom). The loading cell was set to 25 kg and calibrated with a 5-kg standard weight. Force was measured as the compression of one olive. Values in Newtons were recorded as the maximum force detected by the instrument throughout the testing distance of 5 mm at a constant speed of 3 mm/s upon contact of the probe with the olive.

### Enumeration of culturable yeast and bacteria from olive fermentations.

Three olives from each container were placed into separate 207-ml (7-oz) Whirl-Pak filter bags (Nasco, Modesto, CA) containing 3 ml phosphate-buffered saline (PBS) (137 mM NaCl, 2.7 mM KCl, 10 mM Na_2_HPO_4_, 2 mM KH_2_PO_4_ [pH 7.4]) and macerated with a rubber mallet. Approximately 100 µl of the soluble olive macerate suspension was collected from the opposite side of the bag filter for viable cell enumeration. The olive tissue macerate (approximately 500 mg) was frozen at −20°C for DNA extraction as described below. Brine was stirred and collected from the center of each container at a depth of at least 5 cm below the surface. Culturable yeast cells were enumerated by plating serial dilutions of the olive macerate and brine onto Rose Bengal chloramphenicol agar (RBCA) (BD, Franklin Lakes, NJ) supplemented with 100 µg/ml chloramphenicol (Thermo Fisher Scientific, Waltham, MA) to prevent bacterial growth. Bacteria were enumerated on tryptic soy agar (TSA) (Remel, Lenexa, KS) and MRS containing Natacid (25 µg/ml) (Caglificio Clerici, Manzoni, Italy) to prevent yeast and mold growth. When appropriate, Rif was added to MRS at a concentration of 50 μg/ml. MRS agar plates were incubated anaerobically in BD BBL GasPak containers (BD, Franklin Lakes, NJ). All plates were incubated at 30°C for 24 to 48 h prior to colony enumeration.

### PGA activity.

Yeast cells isolated from the brines and olives on RBCA were selected on the basis of colony morphology to test for PGA activity. The yeast isolates were first grown for 24 h in YM containing 1.5% (wt/vol) pectin (Spectrum Chemical Manufacturing Corp., Gardena, CA) and were then dispensed into preformed wells in agar plates containing polygalacturonic acid prepared as previously described (4). The plates were incubated at 30°C for 24 h and stained with 0.05% ruthenium red (Acros Organics, Geel, Belgium) for 30 min, and the PGA activity was quantified by measuring the radius of the zone of clearing around the wells (28). Purified PGA at a concentration of 0.001 mg/ml (Sigma-Aldrich, St. Louis, MO) was used as a positive control.

### DNA extraction, sequencing, and analysis.

Four olives (2 per replicate container) were selected for bacterial and yeast identification. For DNA extraction, 0.1 g of olive macerate was suspended in 180 µl PBS, 100 µl lysis buffer (200 mM NaCl, 100 mM Tris [pH 8], and 20 mM EDTA), and lysozyme (20 mg/ml) in the presence of 0.3 g of 0.1-mm zirconia silica beads (BioSpec Products, Bartlesville, OK) and incubated at 37°C for 30 min. A total of 1.7 ml ASL buffer (QIAamp DNA stool minikit; Qiagen, Valencia, CA) was added prior to lysis in a FastPrep-24 homogenizer (MP Biomedicals, Solon, OH) at a speed setting of 6.5 m/s for 2 min. Finally, the DNA was purified on a QIAamp column (Qiagen) according to the manufacturer’s instructions.

The V4 region of bacterial 16S rRNA genes was amplified from DNA extracted from the olives using barcoded F515 (5′ NNNNNNNNGTGTGCCAGCMGCCGCGGTAA 3′) primers and the R806 primer (5′-GGACTACHVGGGTWTCTAAT-3′) (29). Fungal internal transcribed spacer (ITS) region 1 was amplified by PCR with barcoded forward BITS primer (5′ NNNNNNNNACCTGCGGARGGATCA 3′) and reverse B58S3 primer (5′ GAGATCCRTTGYTRAAAGTT 3′) (30). Each forward primer contained a unique 8-nucleotide bar code (represented by “N”) that enabled multiplexing among samples. For amplification of bacterial 16S rRNA genes, PCR was performed using 5 ng DNA template, 2.5 U GoTaq DNA polymerase (Promega, Madison, WI), 1× Colorless GoTaq reaction buffer, 0.2 mM deoxynucleoside triphosphates (dNTPs) (Promega), 1 mM MgCl_2_, and 5 pmol of each primer. PCR was initiated at 94°C for 3 min, followed by 35 cycles of 94°C for 45 s, 50°C for 1 min, and 72°C for 1.5 min with a final extension at 72°C for 10 min. PCR amplification of fungal ITS was carried out using the same reagents but with 5 pmol of each primer and with PCR conditions set to an initial temperature of 95°C for 2 min followed by 40 cycles of 95°C for 2 min, 55°C for 30 s, and 72°C for 1 min with a final extension at 72°C for 5 min. All amplified products were mixed at similar quantities and purified with the Wizard SV gel and PCR cleanup kit (Promega). The pooled samples were used for library construction and sequenced on an Illumina MiSeq using the paired-end 250-bp protocol at the University of California, Davis Genome Center (http://dnatech.genomecenter.ucdavis.edu/).

Raw 16S rRNA gene and ITS sequence files were analyzed with the QIIME software package version 1.9 (31). Paired-end sequences with a minimum of 50 overlapping nucleotides were joined prior to demultiplexing according to the 8-bp barcode from the forward primers. 16S rRNA gene reads with an average Phred quality score below 30 were discarded prior to chimera checking with USEARCH61 (32). DNA sequences were subsequently clustered into operational taxonomic units (OTUs). GreenGenes database version 13.8, clustered at 97% identity (33, 34), was used for 16S rRNA gene open reference OTU picking and taxonomy assignment.

For ITS sequences, we built a custom yeast reference database on 30 June 2015 (see Table S1 in the supplemental material). This database was constructed because the UNITE database (https://unite.ut.ee/) (30) was not sufficient for identification of yeast taxa known to be present on olives, even after the addition of taxonomy strings to unclassified sequences. For the custom yeast database, ITS sequences of 875 yeast strains were compiled from GenBank (https://www.ncbi.nlm.nih.gov/genbank/) and consisted of yeasts in the 2011 yeast taxonomic treatise (35) as well as new species described after 2011 for which ITS sequences were available. The database also encompassed an additional 9 ITS sequences were obtained from the Portuguese Yeast Culture Collection (http://pycc.bio-aware.com). All sequences differed by at least 1% sequence identity. The database included 164 yeast species known to be associated with olives. Each of the yeast taxonomy strings was edited so that they referenced exactly six taxonomic ranks. Olive-associated species with missing taxonomy strings were manually updated using information from the UniProt database (http://www.uniprot.org/taxonomy). The species with updated taxonomy strings were *Candida boidinii*, *Candida apicola*, *Candida etchellsii*, *Candida parapsilosis*, *Candida pararugosa*, *Candida rugosa*, *Candida solani*, *Pseudozyma graminicola*, and *Pseudozyma shanxiensis*. The custom database served as a reference for OTU picking and taxonomic assignment.

10.1128/mSphere.00315-16.4 TABLE S1  Yeast ITS DNA sequences used for taxonomy assignments. Download TABLE S1, XLSX file, 0.2 MB. Copyright © 2017 Zaragoza et al. 2017 Zaragoza et al. This content is distributed under the terms of the Creative Commons Attribution 4.0 International license.

Bacterial and fungal OTUs with total sequence abundances below 0.00005% were discarded (30) as well as OTUs identified as chloroplast and mitochondria. Three bacterial samples with fewer than 400 sequences and two fungal samples with under 800 sequences were excluded from downstream analyses. For assessments of alpha diversity, bacterial and fungal data were rarefied to sequence depths of 504 and 1,080, respectively, representing the minimum sequence depths among the olive samples. For beta diversity analysis, microbial populations were first normalized using cumulative sum scaling (CSS) because this technique takes undersampling into consideration and was shown to improve clustering analysis of distinct communities (36). Weighted UniFrac (37) distance matrices were generated from the 16S rRNA gene data and subsequently used to produce principal coordinates.

### Statistical analysis.

Statistical differences in pH, salinity, olive firmness, and viable microbial cell numbers between the olive fermentations were calculated with Microsoft Excel (Microsoft, Redmond, WA). For assessments of brine pH and salinity, we aggregated results from days 3 to 76 and from days 106 to 223. We did not include values from the days when acetic acid or salt was added to the fermentations because not all fermentations received those amendments. Olive and brine measurements were tested for heteroscedasticity using Bartlett’s test. Heteroscedastic data were evaluated with Welch’s ANOVA and the Games-Howell *post hoc* test. Homoscedastic data were analyzed using either the Mann-Whitney *U* test or the Kruskal-Wallis test followed by Dunn’s *post hoc* test. Multivariate analysis of taxon enrichment among different treatment groups was performed using the MaAsLin statistical framework (https://huttenhower.sph.harvard.edu/maaslin). Differential taxon abundances between days 37 and 71 were determined with the QIIME differential_abundance.py script employing metagenomeSEQ’s fitZIG algorithm (36). Differences in alpha diversity were calculated with the compare_alpha_diversity.py script using the Student’s *t* distribution test in a pairwise manner with false discovery rate correction.

### Accession number(s).

DNA sequences were submitted to the NCBI Sequence Read Archive (SRA) (https://www.ncbi.nlm.nih.gov/sra) under accession no. PRJNA296398 for both 16S rRNA gene sequences and fungal ITS sequences.

## References

[1] Wolfe BE, Dutton RJ 2015 Fermented foods as experimentally tractable microbial ecosystems. Cell 161:49–55. doi:10.1016/j.cell.2015.02.034. 25815984

[2] Bachmann H, Pronk JT, Kleerebezem M, Teusink B 2015 Evolutionary engineering to enhance starter culture performance in food fermentations. Curr Opin Biotechnol 32:1–7. doi:10.1016/j.copbio.2014.09.003. 25269887

[3] Uylaşer V, Yildiz G 2014 The historical development and nutritional importance of olive and olive oil constituted an important part of the Mediterranean diet. Crit Rev Food Sci Nutr 54:1092–1101. doi:10.1080/10408398.2011.626874. 24499124

[4] Golomb BL, Morales V, Jung A, Yau B, Boundy-Mills KL, Marco ML 2013 Effects of pectinolytic yeast on the microbial composition and spoilage of olive fermentations. Food Microbiol 33:97–106. doi:10.1016/j.fm.2012.09.004. 23122507

[5] Tufariello M, Durante M, Ramires FA, Grieco F, Tommasi L, Perbellini E, Falco V, Tasioula-Margari M, Logrieco AF, Mita G, Bleve G 2015 New process for production of fermented black table olives using selected autochthonous microbial resources. Front Microbiol 6:1007. doi:10.3389/fmicb.2015.01007. 26441932 PMC4585182

[6] Martorana A, Alfonzo A, Settanni L, Corona O, La Croce F, Caruso T, Moschetti G, Francesca N 2016 Effect of the mechanical harvest of drupes on the quality characteristics of green fermented table olives. J Sci Food Agric 96:2004–2017. doi:10.1002/jsfa.7311. 26084955

[7] De Angelis M, Campanella D, Cosmai L, Summo C, Rizzello CG, Caponio F 2015 Microbiota and metabolome of un-started and started Greek-type fermentation of *Bella* *di* *Cerignola* table olives. Food Microbiol 52:18–30. doi:10.1016/j.fm.2015.06.002. 26338113

[8] Grounta A, Doulgeraki AI, Nychas GJ, Panagou EZ 2016 Biofilm formation on *Conservolea* natural black olives during single and combined inoculation with a functional *Lactobacillus* *pentosus* starter culture. Food Microbiol 56:35–44. doi:10.1016/j.fm.2015.12.002. 26919816

[9] Pistarino E, Aliakbarian B, Casazza AA, Paini M, Cosulich ME, Perego P 2013 Combined effect of starter culture and temperature on phenolic compounds during fermentation of *Taggiasca* black olives. Food Chem 138:2043–2049. doi:10.1016/j.foodchem.2012.11.021. 23411341

[10] Abriouel H, Benomar N, Cobo A, Caballero N, Fernández Fuentes MÁ, Pérez-Pulido R, Gálvez A 2012 Characterization of lactic acid bacteria from naturally fermented *Manzanilla Alorena* green table olives. Food Microbiol 32:308–316. doi:10.1016/j.fm.2012.07.006. 22986194

[11] Argyri AA, Nisiotou AA, Mallouchos A, Panagou EZ, Tassou CC 2014 Performance of two potential probiotic *Lactobacillus* strains from the olive microbiota as starters in the fermentation of heat shocked green olives. Int J Food Microbiol 171:68–76. doi:10.1016/j.ijfoodmicro.2013.11.003. 24334091

[12] Blana VA, Grounta A, Tassou CC, Nychas GJ, Panagou EZ 2014 Inoculated fermentation of green olives with potential probiotic *Lactobacillus* *pentosus* and *Lactobacillus plantarum* starter cultures isolated from industrially fermented olives. Food Microbiol 38:208–218. doi:10.1016/j.fm.2013.09.007. 24290645

[13] Rodríguez-Gómez F, Romero-Gil V, Bautista-Gallego J, García-García P, Garrido-Fernández A, Arroyo-López FN 2014 Production of potential probiotic Spanish-style green table olives at pilot plant scale using multifunctional starters. Food Microbiol 44:278–287. doi:10.1016/j.fm.2014.03.023. 25084674

[14] Lanza B 2013 Abnormal fermentations in table-olive processing: microbial origin and sensory evaluation. Front Microbiol 4:91. doi:10.3389/fmicb.2013.00091. 23675370 PMC3650464

[15] Arroyo-López FN, Querol A, Bautista-Gallego J, Garrido-Fernández A 2008 Role of yeasts in table olive production. Int J Food Microbiol 128:189–196. doi:10.1016/j.ijfoodmicro.2008.08.018. 18835502

[16] Hurtado A, Reguant C, Bordons A, Rozès N 2010 Evaluation of a single and combined inoculation of a *Lactobacillus* *pentosus* starter for processing cv. *Arbequina* natural green olives. Food Microbiol 27:731–740. doi:10.1016/j.fm.2010.03.006. 20630314

[17] Hurtado A, Reguant C, Bordons A, Rozès N 2009 Influence of fruit ripeness and salt concentration on the microbial processing of *Arbequina* table olives. Food Microbiol 26:827–833. doi:10.1016/j.fm.2009.05.010. 19835767

[18] Arroyo-López FN, Durán-Quintana MC, Ruiz-Barba JL, Querol A, Garrido-Fernández A 2006 Use of molecular methods for the identification of yeast associated with table olives. Food Microbiol 23:791–796. doi:10.1016/j.fm.2006.02.008. 16943084

[19] Coton E, Coton M, Levert D, Casaregola S, Sohier D 2006 Yeast ecology in French cider and black olive natural fermentations. Int J Food Microbiol 108:130–135. doi:10.1016/j.ijfoodmicro.2005.10.016. 16380183

[20] Botta C, Cocolin L 2012 Microbial dynamics and biodiversity in table olive fermentation: culture-dependent and -independent approaches. Front Microbiol 3:245. doi:10.3389/fmicb.2012.00245. 22783248 PMC3390769

[21] Salma M, Rousseaux S, Sequeira-Le Grand A, Divol B, Alexandre H 2013 Characterization of the viable but nonculturable (VBNC) state in *Saccharomyces cerevisiae*. PLoS One 8:e77600. doi:10.1371/journal.pone.0077600. 24204887 PMC3812164

[22] Oro L, Ciani M, Bizzaro D, Comitini F 2016 Evaluation of damage induced by Kwkt and Pikt zymocins against *Brettanomyces*/*Dekkera* spoilage yeast, as compared to sulphur dioxide. J Appl Microbiol 121:207–214 doi:10.1111/jam.13121. 26939714

[23] Cocolin L, Alessandria V, Botta C, Gorra R, De Filippis F, Ercolini D, Rantsiou K 2013 NaOH-debittering induces changes in bacterial ecology during table olives fermentation. PLoS One 8:e69074. doi:10.1371/journal.pone.0069074. 23935928 PMC3729808

[24] Tataridou M, Kotzekidou P 2015 Fermentation of table olives by oleuropeinolytic starter culture in reduced salt brines and inactivation of *Escherichia coli* O157:H7 and *Listeria monocytogenes*. Int J Food Microbiol 208:122–130. doi:10.1016/j.ijfoodmicro.2015.06.001. 26065729

[25] Maligoy M, Mercade M, Cocaign-Bousquet M, Loubiere P 2008 Transcriptome analysis of *Lactococcus lactis* in coculture with *Saccharomyces cerevisiae*. Appl Environ Microbiol 74:485–494. doi:10.1128/AEM.01531-07. 17993564 PMC2223240

[26] Mendes F, Sieuwerts S, de Hulster E, Almering MJ, Luttik MA, Pronk JT, Smid EJ, Bron PA, Daran-Lapujade P 2013 Transcriptome-based characterization of interactions between *Saccharomyces cerevisiae* and *Lactobacillus delbrueckii* subsp. *bulgaricus* in lactose-grown chemostat cocultures. Appl Environ Microbiol 79:5949–5961. doi:10.1128/AEM.01115-13. 23872557 PMC3811385

[27] Mansour S, Bailly J, Landaud S, Monnet C, Sarthou AS, Cocaign-Bousquet M, Leroy S, Irlinger F, Bonnarme P 2009 Investigation of associations of *Yarrowia lipolytica*, *Staphylococcus xylosus*, and *Lactococcus lactis* in culture as a first step in microbial interaction analysis. Appl Environ Microbiol 75:6422–6430. doi:10.1128/AEM.00228-09. 19684166 PMC2765154

[28] Gregori R, Mari M, Bertolini P, Barajas JAS, Tian JB, Labavitch JM 2008 Reduction of *Colletotrichum* *acutatum* infection by a polygalacturonase inhibitor protein extracted from apple. Postharvest Biol Technol 48:309–313. doi:10.1016/j.postharvbio.2007.10.006.

[29] Caporaso JG, Lauber CL, Walters WA, Berg-Lyons D, Lozupone CA, Turnbaugh PJ, Fierer N, Knight R 2011 Global patterns of 16S rRNA diversity at a depth of millions of sequences per sample. Proc Natl Acad Sci U S A 108(Suppl 1):4516–4522. doi:10.1073/pnas.1000080107. 20534432 PMC3063599

[30] Bokulich NA, Mills DA 2013 Improved selection of internal transcribed spacer-specific primers enables quantitative, ultra-high-throughput profiling of fungal communities. Appl Environ Microbiol 79:2519–2526. doi:10.1128/AEM.03870-12. 23377949 PMC3623200

[31] Caporaso JG, Kuczynski J, Stombaugh J, Bittinger K, Bushman FD, Costello EK, Fierer N, Peña AG, Goodrich JK, Gordon JI, Huttley GA, Kelley ST, Knights D, Koenig JE, Ley RE, Lozupone CA, McDonald D, Muegge BD, Pirrung M, Reeder J, Sevinsky JR, Turnbaugh PJ, Walters WA, Widmann J, Yatsunenko T, Zaneveld J, Knight R 2010 QIIME allows analysis of high-throughput community sequencing data. Nat Methods 7:335–336. doi:10.1038/nmeth.f.303. 20383131 PMC3156573

[32] Edgar RC 2010 Search and clustering orders of magnitude faster than BLAST. Bioinformatics 26:2460–2461. doi:10.1093/bioinformatics/btq461. 20709691

[33] DeSantis TZ, Hugenholtz P, Larsen N, Rojas M, Brodie EL, Keller K, Huber T, Dalevi D, Hu P, Andersen GL 2006 Greengenes, a chimera-checked 16S rRNA gene database and workbench compatible with ARB. Appl Environ Microbiol 72:5069–5072. doi:10.1128/AEM.03006-05. 16820507 PMC1489311

[34] McDonald D, Price MN, Goodrich J, Nawrocki EP, DeSantis TZ, Probst A, Andersen GL, Knight R, Hugenholtz P 2012 An improved Greengenes taxonomy with explicit ranks for ecological and evolutionary analyses of bacteria and archaea. ISME J 6:610–618. doi:10.1038/ismej.2011.139. 22134646 PMC3280142

[35] Kurtzman C, Fell JW, Boekhout T 2011 The yeasts: a taxonomic study, 5th ed. Elsevier, Philadelphia, PA.

[36] Paulson JN, Stine OC, Bravo HC, Pop M 2013 Differential abundance analysis for microbial marker-gene surveys. Nat Methods 10:1200–1202. doi:10.1038/nmeth.2658. 24076764 PMC4010126

[37] Lozupone C, Knight R 2005 UniFrac: a new phylogenetic method for comparing microbial communities. Appl Environ Microbiol 71:8228–8235. doi:10.1128/AEM.71.12.8228-8235.2005. 16332807 PMC1317376

