# Supplementary figures and images for: Effects of Exogenous Yeast and Bacteria on the Microbial Population Dynamics and Outcomes of Olive Fermentations
Source: mSphere. 2017 Jan 18;2(1):e00315-16. doi: 10.1128/mSphere.00315-16 (PMC5244262; doi:10.1128/mSphere.00315-16)

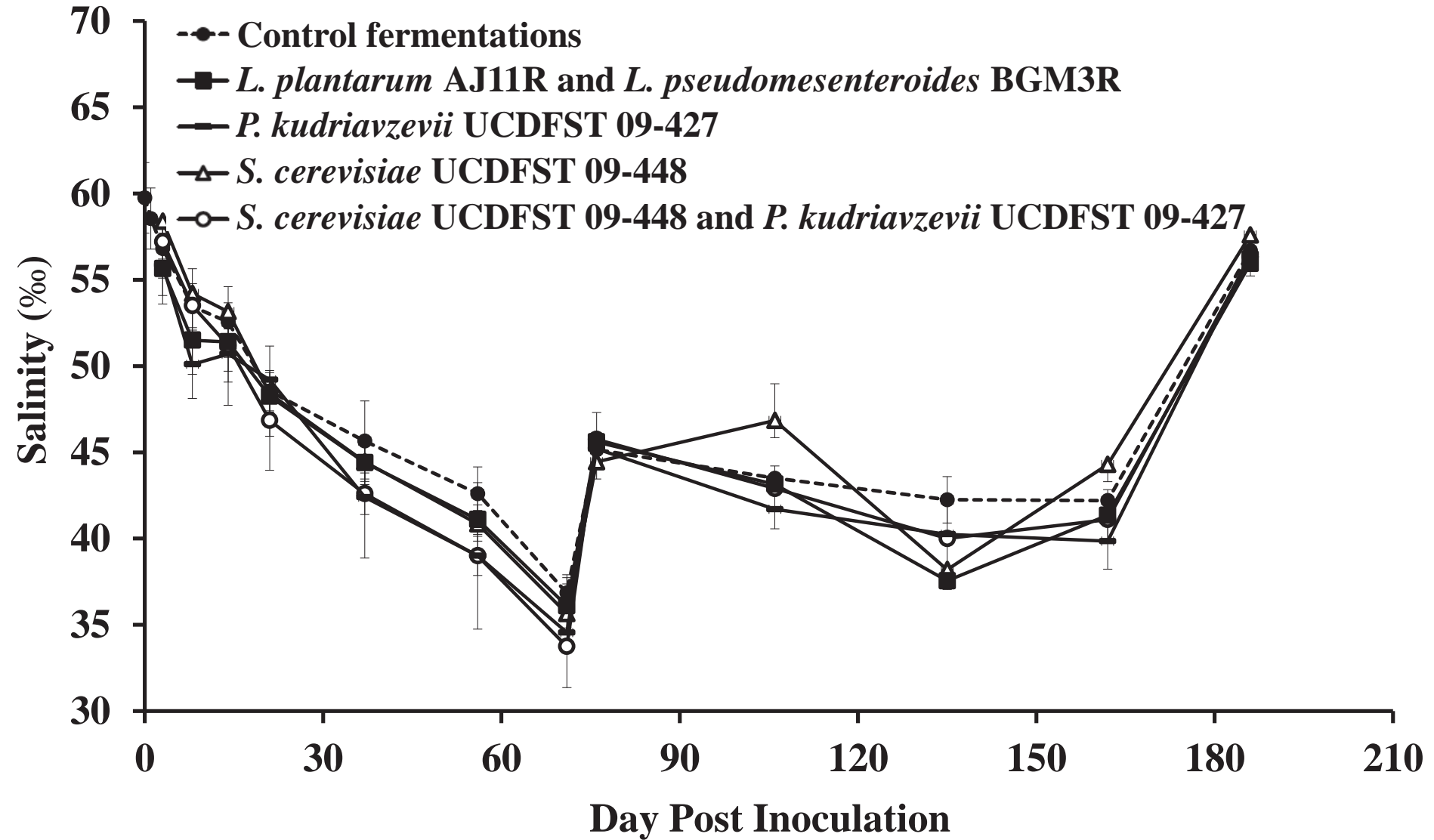

Supplement: FIG S2 [file sph001172226sf3.pdf]

Relative Abundance

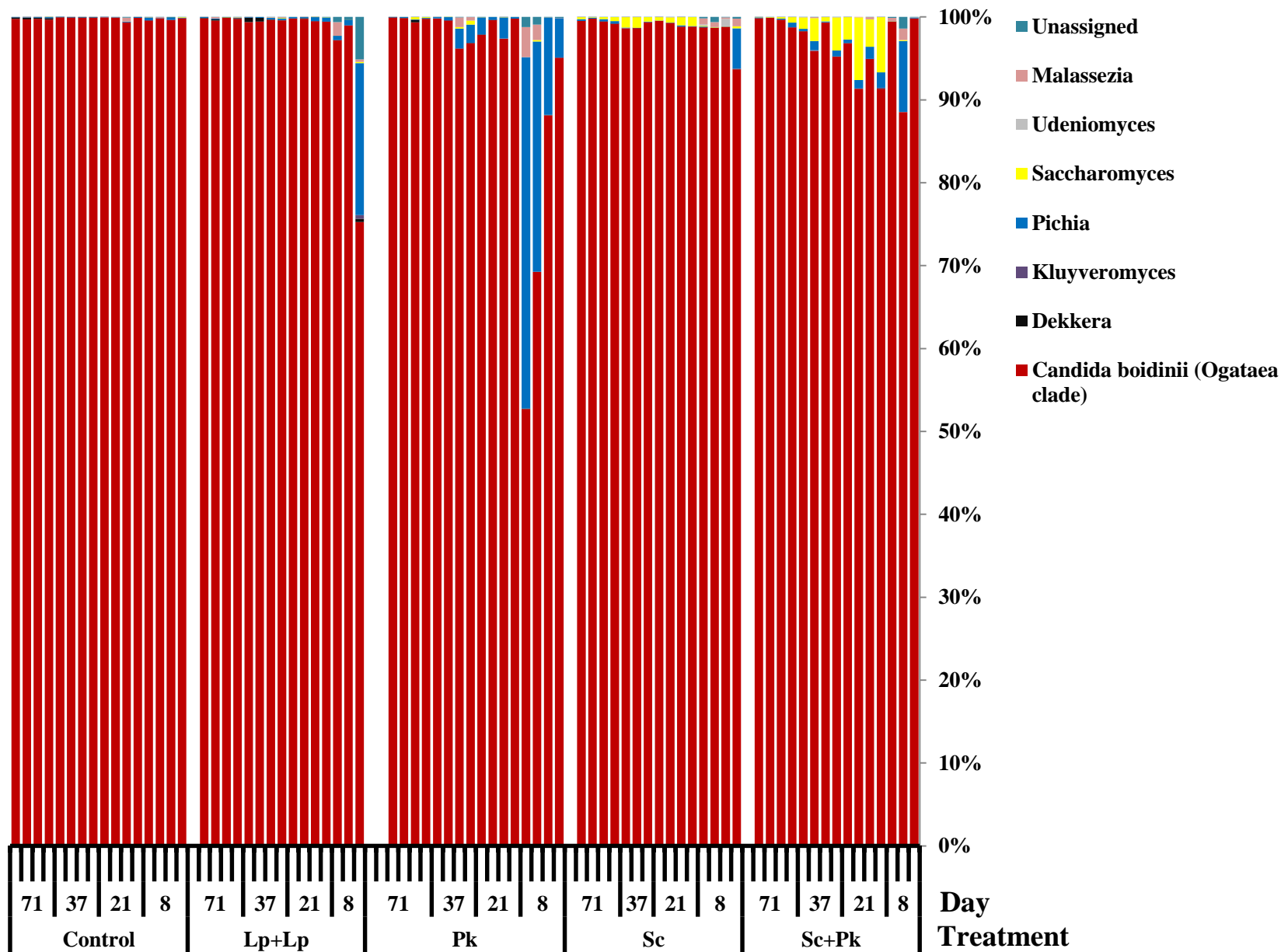

Supplement: FIG S3 [file sph001172226sf4.pdf]
